# Assessment of total and regional bone mineral density using bioelectrical impedance vector analysis in elderly population

**DOI:** 10.1038/s41598-021-00575-1

**Published:** 2021-10-27

**Authors:** Hsueh-Kuan Lu, Chung-Liang Lai, Li-Wen Lee, Lee-Ping Chu, Kuen-Chang Hsieh

**Affiliations:** 1 General Education Center, National Taiwan University of Sport, Taichung, 404 Taiwan, ROC; 2 Department of Occupational Therapy, Asia University, Taichung, 413 Taiwan, ROC; 3 Department of Diagnostic Radiology, Chang Gung Memorial Hospital, Chiayi, 613 Taiwan, ROC; 4 Department of Orthopedics, China Medical University Hospital, Taichung, 404 Taiwan, ROC; 5 Big Data Center, National Chung Hsing University, Taichung, 402 Taiwan, ROC

**Keywords:** Medical research, Predictive markers

## Abstract

This study aimed to investigate the relationship between bone mineral density (BMD) and height-adjusted resistance (R/H), reactance (Xc/H) and phase angle (PhA). A total of 61 male and 64 female subjects aged over 60 years were recruited from middle Taiwan. The R and Xc were measured using Bodystat Quadscan 4000 at a frequency of 50 kHz. BMD at the whole body, L2–L4 spine, and dual femur neck (DFN), denoted as BMD_Total_, BMD_L2–L4,_ and BMD_DFN_, were calculated using a Hologic DXA scanner. The R-Xc graph was used to assess vector shift among different levels of BMD. BMD was positively correlated with Xc/H and negatively correlated with R/H (*p* < 0.001). The General Linear Model (GLM) regression results were as follows: BMD_Total_ = 1.473–0.002 R/H + 0.007 Xc/H, *r* = 0.684; BMD_L2–L4_ = 1.526–0.002 R/H + 0.012 Xc/H, *r* = 0.655; BMD_DFN_ = 1.304–0.002 R/H + Xc/H, *r* = 0.680; *p* < 0.0001. Distribution of vector in the R-Xc graph was significantly different for different levels of BMD_Total_, BMD_L2–L4_ and BMD_DFN_. R/H and Xc/H were correlated with BMD in the elderly. The linear combination of R/H and Xc/H can effectively predict the BMD of the whole body, spine and proximal femur, indicating that BIVA may be used in clinical and home-use monitoring tool for screening BMD in the elderly in the future.

## Introduction

The bones act as a supportive structure for the human body. The mineral content of human bone tissue provides bone its architecture and strength^1^. Bone strength is directly related to bone mineral density (BMD) and a decrease in BMD is associated with increased fracture risk^2^. Changes in bone mineral content (BMC) and BMD are age- and gender-dependent. With aging, BMC and BMD increase with increasing age, reaching a maximum at the 20s, and then slowly decline over time in both genders^3^. In postmenopausal women, a low level of estrogens further accelerates bone loss. For women, the overall lifelong decrease in BMD ranges up to 58% in the femoral neck and 42% in the spine whereas the rate of decrease in BMD was two-thirds of that in women for femoral neck and only one-fourth of that in women for the lumbar spine than men^4^. Therefore, women also have a higher risk of osteoporotic fracture than men. The lifetime risk of fracture is estimated to be 44% for women aged 60 years old while the risk is 25% for the men^5^. Osteoporotic fracture is a serious health issue that can lead to dependency, morbidity, and mortality. With the increasing trend in life expectancy, the total number and associated costs of osteoporotic fractures continue to rise globally.

Osteoporosis affects the skeleton disproportionately. Thus, BMD should be measured at specific fracture sites of interest such as the spine, proximal femur, and forearm for accurate risk assessment. Common bone densitometries include dual-energy X-ray absorptiometry (DXA), quantitative CT (QCT), and quantitative ultrasound (QUS)^6^. DXA is considered the gold standard method in clinical practice. A DXA scanner uses two different energies of X-ray to partition the body mass into BMC and soft tissue and calculate the corresponding BMD. However, the DXA scanner has the disadvantages of high cost and limited availability and therefore may not be suitable for large scale study.

Bioelectrical impedance analysis (BIA) can be used to calculate body composition based on the electrical properties of the body, assuming the measured body part is a cylindrical shape with a fixed cross-sectional area, the body composition is homogeneous, the distribution of current density is consistent and the body is under stable hydration status without water–electrolyte imbalance. Violation of the prior assumptions may cause errors in measurement. Currently, BIA is a well-known method for calculating body composition in clinical practice and field studies owing to its portability, low cost, and high precision. The mineral content is a less established BIA estimate which can be obtained from modern BIA. The performance for predicting BMC using mineral estimates by BIA was reported to be high in correlation but with the limited agreement in healthy young adults^7^.

The phase shift is defined as the phase angle (PhA), which is a BIA parameter directly calculated from the BIA electrical components of resistance (R) and reactance (Xc). Since the human cell membrane is a phospholipid bilayer, forming a double-layered sheet with the hydrophilic end outside and hydrophobic layer inside, it can behave as a capacitor. In a resistor/capacitor circuit connected to an AC voltage, there is a phase difference between current and voltage sinusoidal waveform. Resistance quantifies the opposition to the flow of a current through biological fluids and tissue. All in all, the reactance (Xc) depends on the integrity and quantity of the cell membrane, while the resistance (R) is related to the total body fluid and ion composition. Therefore, vector components represent cellular mass and function in bioelectrical impedance vector analysis (BIVA). At a frequency of 5–50 kHz, the current passes through an extracellular fluid but not intracellular fluid. Therefore, most BIA devices measure PhA at a frequency of 50 kHz. PhA results depend on several biological factors such as cell mass, integrity, and permeability of cell membrane, and the amount of fluid in the intracellular and extracellular spaces. Unlike the body composition estimates by BIA which are calculated via the application of empirical equations, PhA is an assumption-free raw BIA parameter and therefore PhA is less biased and can be applied in patients with different hydration status. The applications of PhA attract researchers attention and have been applied to study muscle quality and muscle strength^8–11^, sarcopenia^12^, physical performance^13^, inflammatory markers^14^, nutrition status^15^, disease prognosis^16^ and risk of mortality^17^. A recent study by Antunes et al*.*^18^ reported that PhA is correlated to BMD in the elderly population, launching a new application of PhA in studying BMD.

The bioelectrical impedance vector analysis (BIVA) method was proposed in 1994 by Piccoli et al*.*^19^ by plotting 2-dimensional vectors on height-standardized R and Xc axes, which is called the R-Xc graph. Alternatively, any point in the 2-dimensional space of the R-Xc graph can be described by the position vector with length and angle components. Vector shift due to low or high Xc indicates a decrease or increase of dielectric mass (cell membranes and tissue interfaces)^20^. Change in vector length can be used to represent hydration status, with lengthening of the vector indicates fluid loss and shortening of the vector indicates fluid gain. The concept is an important precursor for BIVA to provide information on body cell mass, cell integrity, and hydration status using vector length and distribution. Currently, BIVA has been used to assess the hydration and nutritional status in hemodialysis patients^21,22^, liver cirrhosis patients^23^, critically ill patients^24^, obese adults^25^, and nutritional status of healthy adults^26^.

PhA has been increasingly used in nutritional and clinical disease assessment and BIVA has been used to evaluate water content, nutritional status, and skeletal muscle function. However, there are only few studies regarding the application of PhA in estimating BMD^18^. This study was aimed to investigate the relationship between BIVA parameters and BMD in total body and body segments in the elderly population. The vector shift in the R-Xc graph was also examined.

## Results

Table 1 shows descriptive statistics of anthropometric and bioelectrical and body composition variables by gender. There was no significant difference in the age and BMI between men and women. However, the percentage body fat of male subjects was significantly lower than that of female subjects, and the BMD of male subjects in all different regions tested was significantly higher than that of female subjects (*p* < 0.001). In addition, the values of Z, R, Xc, R/H, Xc/H and PhA were all significantly different between genders (*p* < 0.001).

**Table 1:** Subject demographic.

	Total (n = 125)	Female (n = 64)	Male (n = 61)
Age (year)	65.97 ± 4.84 (60.0, 83.4)	66.67 ± 5.63 (60.0, 83.4)	65.22 ± 3.72 (60.7, 74.5)
Height (m)	1.58 ± 0.10(1.43, 1.83)	1.52 ± 0.05 (1.43, 1.66)	1.66 ± 0.09 (1.50, 1.83)**
Weight (kg)	65.76 ± 16.25 (39.70, 105.8)	58.60 ± 12.66 (39.70, 93.42)	73.54 ± 16.21 (43.80, 105.8)**
BMI (kg/m^2^)	25.85 ± 4.73 (16.48, 41.60)	25.44 ± 5.49 (16.48, 41.60)	26.29 ± 3.75 (19.47, 34.2)
Z (ohm)	527.1 ± 95.5 (373.9, 765.9)	589.33 ± 65.2 (454.2, 765.9)	459.6 ± 75.4 (373.9, 739.5)**
R (ohm)	524.2 ± 95.6 (370, 762)	586.4 ± 65.0 (452.0, 762.0)	456.7 ± 75.5 (370.0, 737.0)**
Xc (ohm)	54.4 ± 8.1 (40, 77)	57.85 ± 7.86 (40.0, 77.0)	50.67 ± 6.50 (41.0, 62.0)**
R/H (ohm/m)	333.6 ± 73.6 (226.8, 508.0)	386.47 ± 42.07 (297.4, 508.0)	276.22 ± 55.04 (226.8, 491.3)**
Xc/H (ohm/m)	34.5 ± 6.0 (23.3, 51.3)	38.1 ± 5.7 (26.9, 51.3)	30.6 ± 4.3 (23.3, 40.7)**
PhA (°)	6.04 ± 0.91 (4.41, 8.97)	5.65 ± 0.62 (4.41, 7.0)	6.42 ± 1.01 (4.73, 9.0)**
BF% (%)	38.2 ± 10.3 (17.1, 56.1)	45.2 ± 7.5 (25.5, 56.1)	30.7 ± 7.1 (17.1, 43.1)**
BMD_Total_ (g/cm^2^)	1.19 ± 0.14 (0.92, 1.60)	1.11 ± 0.09 (0.92, 1.41)	1.28 ± 0.14 (1.00, 1.60)**
BMD_L2–L4_ (g/cm^2^)	1.26 ± 0.19 (0.93, 1.72)	1.18 ± 0.14 (0.93, 1.65)	1.35 ± 0.20 (0.97, 1.72)**
BMD_DFN_ (g/cm^2^)	0.99 ± 0.18 (0.66, 1.480)	0.89 ± 0.11 (0.66, 1.28)	1.10 ± 0.17 (0.76, 1.48) **

*PhA* phase angle, *BF%* percentage body fat.

*p < 0.05.

**p < 0.001.

^a^Data are expressed as mean ± standard deviation (min, max).

^b^Z, impedance, R, resistance, Xc, reactance, R/H, resistance standardized for height, Xc/H, reactance standardized for height.

^c^Subscript total, L2–L4, and DFN denote whole body, AP spine L2–L4, and dual femur neck, respectively.

Multiple regression analysis was used to predict BMD_total_, BMD_L2–L4_, and BMD_DFN_ by R/H and Xc/H. Table 2 shows the results of the GLM analysis, including regression weights (coefficients) and the correlation between predicted and actual BMD values. All three regions of BMD estimates were positively correlated with Xc/H and negatively correlated with R/H. The overall models were significant r (*p* < 0.001) with moderate correlation (*r* = 0.655–0.684), indicating a linear combination of R/H and Xc/H predicted BMD in the whole body, spine, and proximal femur.

**Table 2:** Results from the GLM regression analysis showing similar associations between resistance and reactance standardized for height and bone mineral density.

	Constants	Coefficients		r	p (model)
		R/H	Xc/H		
BMD_Total_	1.4732	− 0.0016**	0.0066*	0.684	< 0.0001
BMD_L2–L4_	1.5259	− 0.0021**	0.0121*	0.655	< 0.0001
BMD_DFN_	1.3044	− 0.0019**	0.0093*	0.680	< 0.0001

All distributions of residuals were compatible with a normal distribution.

*p < 0.05.

**p < 0.001.

**Figure 1:**
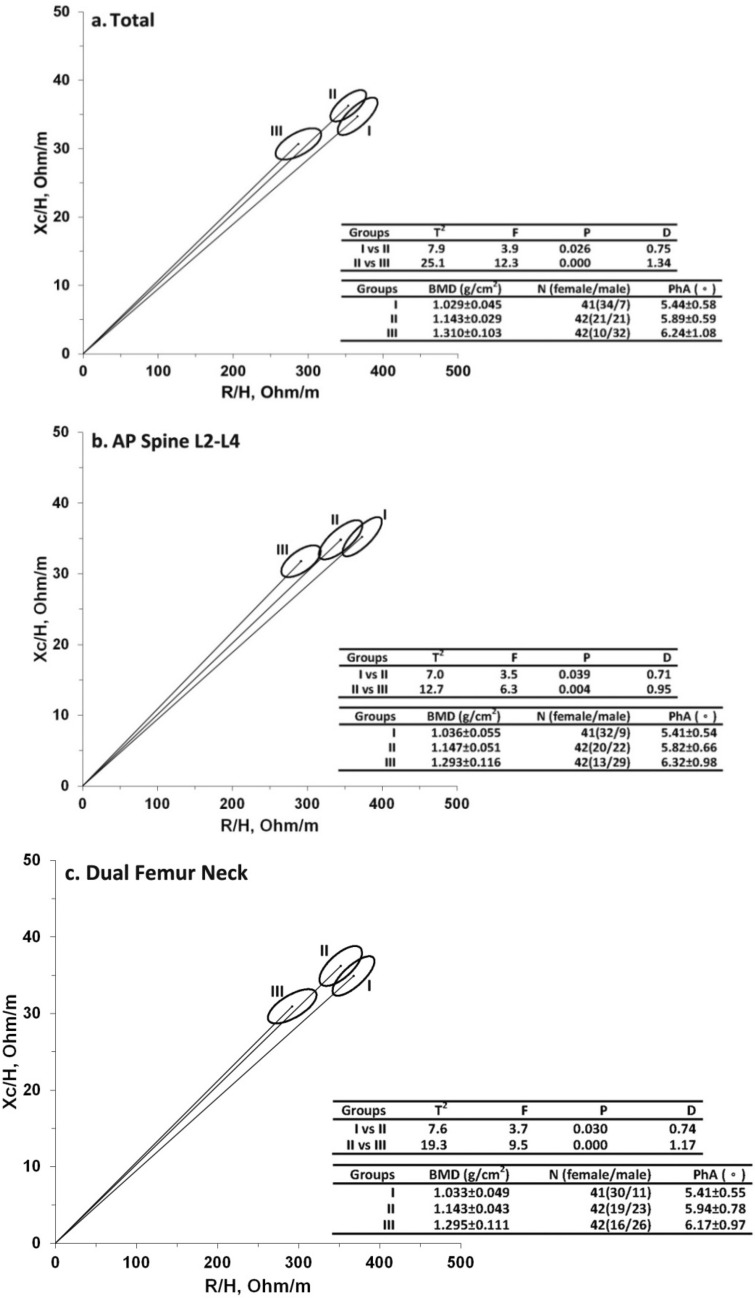
Mean graph with 95% confidence interval ellipse. (**a**). BMD-Total; (**b**) BMD-AP Spine L2–L4; (**c**) BMD-Dual Femur Neck; Hotelling’s T^2^ test value with the corresponding F test and P values, and the Mahalanobis’ generalized distance D. *PhA* phase angle, *Group I* participants with the lowest BMD level, *Group II* participants with middle BMD level, *Group III* patients with the highest BMD level.

Subjects were then divided into three subgroups regardless of gender, according to BMD levels: the lowest (group I), middle (group II) and highest (group III) tertiles of BMD. The R-Xc graph and 95% confidence ellipses of the BMD_total_, BMD_L2–L4,_ and BMD_DFN_ for the three subgroups were shown in Fig. 1a–c, respectively. The results of Hotelling’s T^2^ test showed a significant vector displacement between the BIVA measured in the three subgroups in the whole body, spine, and proximal femur (*p* < 0.05).

**Figure 2:**
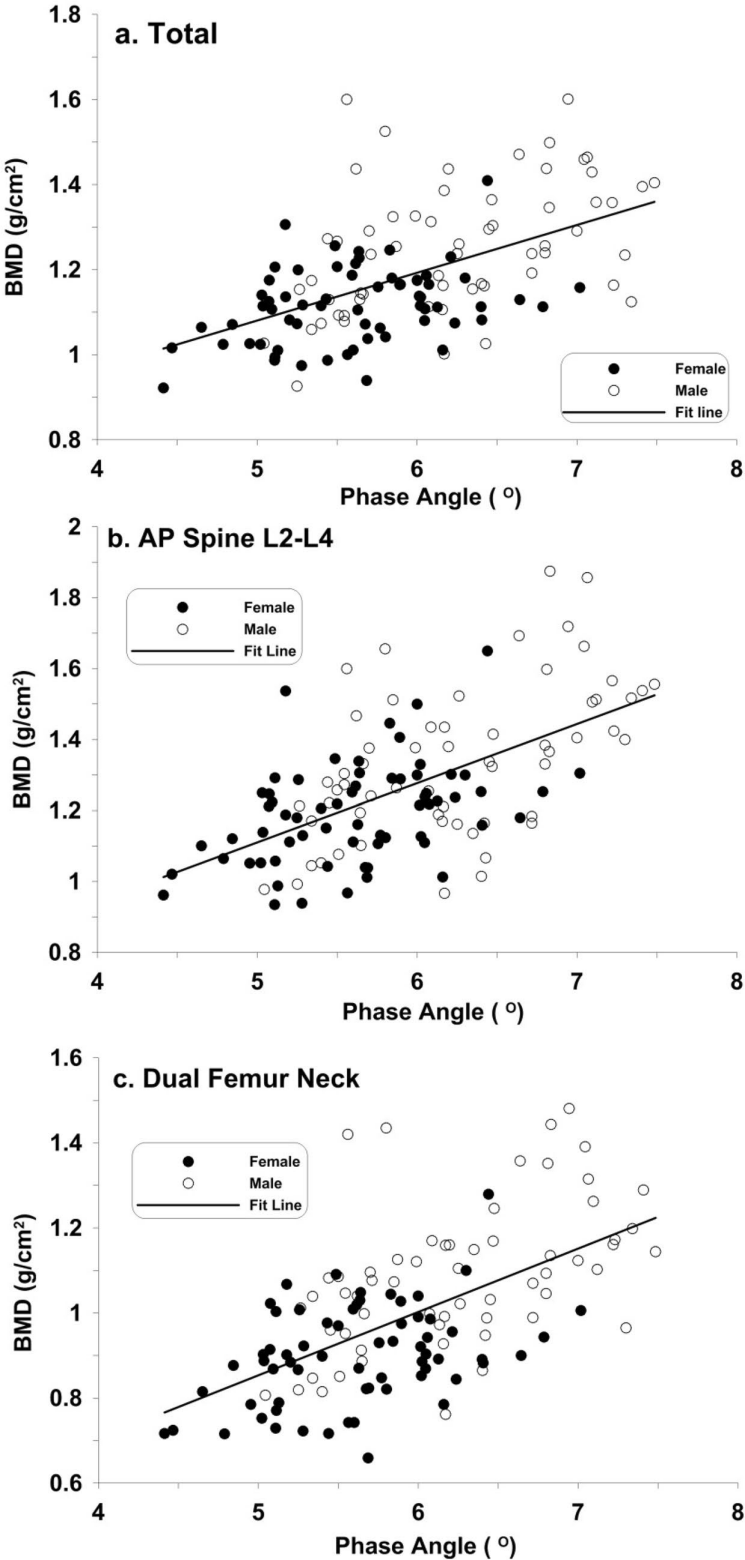
Scatter diagram of phase angle and bone density. (**a**) Total (BMD_total_ = 0.519 + 0.112 PhA, r = 0.538); (**b**) AP Spine L2–L4 (BMD_L2–L4_ = 0.277 + 0.155 PhA, r = 0.582); (**c**) Dual Femur Neck (BMD_FDN_ = 0.108 + 0.148 PhA, r = 0.590).

Scatter plots with linear regression lines of BMD versus PhA in the whole body, spine, and proximal femur were shown in Fig. 2a–c, respectively. In the three anatomical locations, there were moderately positive correlations between BMD and PhA (*r* = 0.538–0.590).

## Discussion

This study showed that BIVA outcomes including PhA, height-adjusted R (R/H), and Xc (Xc/H), were related to BMD levels in the whole body, spine, and proximal femur in the elderly population. This provided a new biomarker for BMD using BIVA. To our knowledge, only one study has related PhA as a BMD determinant and no study has related vector shift in the RXc graph with the changes in BMD. In the study by Antunes et al.^18^, a significant correlation (p < 0.001) was found between PhA and BMD in the total body, femur, femoral neck, and forearm with correlation coefficients of 0.475, 0.524, 0.450, and 0.437, respectively, but no correlation was found in the spine. The cause of the discrepancy remains unclear. This study is consistent with Antunes et al. showing a moderately positive correlation between BMD and PhA.

The phase angle is the most established BIVA parameter. It is considered to be age- and gender-dependent^27^. When adding body composition parameters as variables, age explained most of the variability in PhA, followed by the fat-free mass (FFM) in healthy subjects^28^. In a population study, PhA was found to be associated with frailty and mortality, independent of age and comorbidity^17,29^. Additionally, FFM is positively correlated with BMD with varying strength of the correlation, depending on the sites of BMD^30,31^. Therefore, it is reasonable to expect that PhA may be correlated with BMD. However, more research is needed to explore whether potential confounding variables such as age, gender, height, and weight can affect the correlational relationship between PhA and BMD in different body regions.

R and Xc are BIVA parameters directly measured from the BIA device. By normalized to body height, R/H and Xc/H can provide a qualitative measure of soft tissue that does not depend on body size^20^. This study showed that BMD was positively correlated with Xc/H but negatively correlated with R/H. Each unit increased in Xc/H (Ohm/m) was associated with 0.0066 g/cm^2^ increase in BMD_total_ whereas each unit increased in R/H (Ohm/m) was associated with a 0.0016 g/cm^2^ decrease in BMD_total_. A similar trend was observed for estimating BMD in the spine and proximal femur in this study. This is the first study suggesting that BIVA variables might be predictors for BMD in the elderly population. Interestingly, this trend has also been observed in previous studies where muscle strength was positively correlated with Xc/H and negatively correlated with R/H^8,10^. Muscle strength is a positive predictor for BMD^32,33^, and this might explain why R/H and Xc/H predict both muscle strength and BMD in a similar direction.

In the BIVA plot, a vector has angle as well as magnitude information in the R/H and Xc/H directions. In contrast, a PhA only has the directional component without providing information on its distance. Indeed, vectors of varying length share the same PhA in the RXc graph. Therefore, the vector analysis approach has the potential of presenting more diverse information on biological activity compared to PhA alone. In addition, the BIA device is a reliable tool, providing measurement with a precision error of 2–3%^34,35^. Currently, BIVA has been used to differentiate obesity (higher PhA and short length), athletes (higher PhA, long length), cachexia (lower PhA, long length), and weakens (normal PhA and short length). In this study, higher BMD was associated with a vector of higher PhA and shorter length. This may provide a diagnostic tool for estimating BMD in the elderly population at risk of osteoporosis.

The present study showed that PhA, R/H, and Xc/H estimates were correlated with BMD. A low PhA is associated with osteoporosis, despite age and gender control^18,36^. BIA device is well-known for personal body fat monitoring at home. However, the current home-use models do not provide BIVA estimates. With the development of home-use BIA devices providing BIVA estimates, a self-screening tool for osteoporosis may be created, aiding in the early diagnosis and treatment of osteoporosis.

Studies have shown that skeletal muscle mass^37,38^ and strength^39,40^ are correlated with BMD. Since BIA parameters can be used as an alternative to muscle mass and muscle strength^8,10^, it is reasonable that there is a correlation between skeletal muscle mass and strength with BMD. Although BIA estimates skeletal muscle mass and strength indirectly using electrical parameters, it is a still a valuable tool due to its high precision and low cost and could facilitate the evaluation of BMD in field studies.

This study has several limitations. First, a cause-effect relationship between BMD and height-adjusted R and Xc could not be established. Second, subgroup analysis was not performed due to the small sample size. Third, osteoporosis is a complex disease governed by a wide range of phenotypes regulated by genetic and environmental factors^41^. However, these factors such as age, gender, ethnicity, family history, physical activity were not included as variables in the regression analysis. Further study is still needed to investigate the impact of these factors on BMD.

## Methods

### Participants

This is a cross-sectional study conducted between January 2016 and December 2016 at the Department of Rehabilitation of the Taichung Hospital in Taiwan. Male and female subjects aged above 60 years were recruited via outpatient clinics at the Department of Rehabilitation of the Taichung Hospital. Written informed consent was obtained from all participants after explaining the study. Subjects interested in participating underwent a screening process by trained health professionals to determine if they qualify for the study. Exclusion criteria were subjects with electrolyte imbalance, cancer, inflammation (serum TNFα ≥ 0.753–1.660 pg/mL or IL-6 ≥ 7 pg/mL), severe cardiovascular disease, and lung disease. A total of 61 males (aged 65.2 ± 3.7 years) and 64 females (66.7 ± 5.6) who met the criteria were scheduled for the measurements. Before the test, participants were asked to start their 8-h fast after breakfast, abstain from alcohol and avoid vigorous exercise for 48 h, and diuretics for 7 days. Before taking the measurements, participants were asked to remove all cognitive content from their body and empty their bladder. All measurements were performed with participants wearing light clothes and no shoes at room temperature (26–28 °C). The total study time was 1–1.5 h on the same afternoon section.

### Ethics statement

The study protocol was approved by the Institutional Review Board of the Tsaotun Psychiatric Center (IRB_1050125) and was registered on the Chinese Clinical Trial Registry (ChiCTR-OOC-16008825). The investigation was conducted in accordance with the 2008 version of the Declaration of Helsinki.

### Body index measurements

Weight was recorded using an electrical scale (Tanita BC418MA, Japan) adjusted to the nearest 0.1 kg and height was measured using a stadiometer (Holtain Ltd, Crosswell, Wales, UK) adjusted to the nearest 0.5 cm. Body mass index (BMI) was calculated by divided weight in kilograms by height in meter squared.

### Bioelectrical impedance analysis measurements

The QuadScan 4000 (Bodystate Ltd, British Islands) was used to record BIA parameters with QuadScan medical graded electrodes. The instrument was calibrated at the beginning of each day of use with a calibrator supplied with the device. A frequency of 50 kHz was used in the hand-to-foot model with participants in a supine position after lying down for at least 10 min. A pair of measuring and current electrodes were placed on the dorsal side of the right hand and another pair on the dorsal surface of the right foot with a 5 cm distance apart at the same anatomical site. Parameters including resistance (R) and reactance (Xc) were measured. Impedance (Z) was calculated as $Z=\sqrt{{R}^{2}+{Xc}^{2}}$. Phase angle (PhA) was calculated as $PhA=arctan (Xc/R)\times {180}^{^\circ }/\pi$. For BIVA, R and Xc were normalized to height (H) and the R-Xc graph was generated by plotting Xc/H versus R/H.

### Dual-energy X-ray absorptiometry measures

Bone estimates were measured using a fan-beam Discovery W scanner (Hologic Inc. Bedford, MA, USA) equipped with software version 5.67. The scanner generates two different energies of X-ray, 100 and 140 kVp, to calculate the BMC and soft tissue composition in the scanned region. Before each day of use, a daily machine calibration test according to the manufacturer’s instructions was performed using the Hologic spine and whole-body phantoms. For the DXA experiment, participants were positioned in the scanning table, according to manufacturer guidelines, by experienced radiological technicians. BMD at the whole body, lumbar spine, and proximal femur were denoted as BMD_total_, BMD_L2–L4_ and BMD_DFN_. The coefficients of variation were 0.5%, 1.1%, and 0.9% for BMD_total_, BMD_L2–L4,_ and BMD_DFN_, respectively.

### Statistical analysis

Data were presented as mean, standard deviation, minimum, and maximum. Statistical analysis was performed using SPSS version 21 (SPSS Inc., Chicago, IL, USA). Shapiro–Wilk test was used to test if a variable was normally distributed. Student t-test was used to determine statistical difference between gender in the variables. ANOVA was used to compare the means between two or more groups of variables. Pearson correlation coefficient was applied to investigate the relationship between two continuous variables. A General Linear Model (GLM) was applied to fit regression models of BMD in different regions using BIVA parameters (R/H and Xc/H).

A value of p < 0.05 was considered statistical significance. BIVA Software 2002^42^ was used to plot the R-Xc graph and confidence ellipses for mean vectors. The 95% confidence ellipses did not overlap was considered statistical significance. The Mahalanobis’s generalized distance (D) was used to rank the difference among mean vectors. Hotelling’s T^2^ test was a multivariate extension of the Student’s t-test in comparison of mean vectors.
